# Adoption of intravascular imaging-guided percutaneous coronary intervention in the United States (2012–2022): temporal trends, disparities, and mortality association

**DOI:** 10.3389/fcvm.2026.1930050

**Published:** 2026-10-07

**Authors:** Yangyang Zhang, Mingyou Zhang, Tong Li, Guowei Zhang, Zheng Xu

**Affiliations:** 1 Department of Cardiovascular Diseases, The First Hospital of Jilin University, Changchun, China; 2 Department of Cardiology, Dehui City People’s Hospital, Changchun, China

**Keywords:** healthcare disparities, intravascular imaging, intravascular ultrasound, mortality, national inpatient sample, percutaneous coronary intervention, time trends, United States

## Abstract

**Background:**

Intravascular imaging (IVI)-guided percutaneous coronary intervention (PCI), using intravascular ultrasound (IVUS) or optical coherence tomography (OCT), improves clinical outcomes. Contemporary data on US adoption trends, demographic, and hospital disparities remain limited.

**Methods and results:**

We analyzed 5,220,590 PCI admissions from the National Inpatient Sample (2012–2022). IVI use was defined by concurrent procedural codes for IVUS or OCT. Outcomes included temporal trends, in-hospital mortality, resource utilization, and disparities. Given the large sample size, standardized mean differences (SMDs) were reported to quantify between-group differences, with an absolute SMD of >0.10 indicating a meaningful imbalance. Multivariable logistic regression was adjusted for demographics, comorbidities, and hospital characteristics. To further address potential confounding by indication, propensity score matching was performed. Patients were matched 1:1 using nearest-neighbor matching without replacement, with a caliper of 0.2 standard deviations of the propensity score and exact matching on calendar year. Covariate balance before and after matching was assessed using absolute SMDs, with an absolute SMD <0.10 indicating an adequate balance. IVI-guided PCI increased from 7.1% (2012) to 21.3% (2022) (*p*_trend_ < 0.001). Minimal demographic disparities existed, but significant hospital-level variations were observed; hospitals in the Northeast had the highest adoption (27.6% in 2022 vs. 16.3% in Western hospitals). In-hospital mortality occurred in 2.32% of IVI hospitalizations compared with 2.88% of hospitalizations without IVI guidance. After adjusting for demographic characteristics, acute clinical presentation and severity—including STEMI, NSTEMI, cardiogenic shock, and cardiac arrest—comorbidities, payer, hospital characteristics, and calendar year, IVI remained significantly associated with lower odds of in-hospital mortality (aOR 0.783, 95% CI 0.742–0.827; *P* < 0.001), higher routine discharge rates (84.1% vs. 83.7%; *p* < 0.001), longer median length-of-stay (3 days [interquartile range (IQR) 2–5] vs. 2 days [IQR 2–4]; *p* < 0.001), and increased costs [$25,144 (IQR $18,749–$36,439) vs. $20,165 (IQR $15,293–$28,381); *p* < 0.001].

**Conclusion:**

While IVI-guided PCI adoption increased significantly, pronounced hospital-level disparities exist. Targeted efforts to enhance adoption—particularly in rural, non-teaching hospitals and low-utilization regions—are needed.

## Background

Percutaneous coronary intervention (PCI) is a cornerstone in managing coronary artery disease (CAD). While angiography guides stent placement, its limitations in assessing vessel morphology and optimal stent deployment are well established. Intravascular imaging (IVI)—comprising intravascular ultrasound (IVUS) and optical coherence tomography (OCT)—provides high-resolution coronary visualization, enabling plaque characterization, precise vessel sizing, and detection of suboptimal stent implantation to optimize PCI.

Mounting evidence confirms the superiority of IVI-guided PCI over angiography alone (1–8). A network meta-analysis of 22 randomized trials (*n* = 15,964) demonstrated a significant reduction in cardiac death, stent thrombosis, and target lesion revascularization. Correspondingly, the 2024 ESC guidelines for managing chronic coronary syndromes upgraded IVI guidance to Class Ia for PCI recommendations when performing PCI on anatomically complex lesions, left main stem, bifurcations, and long lesions (9). Similarly, the 2025 ACC/AHA/ACEP/NAEMSP/SCAI Guideline for managing patients with acute coronary syndromes (10) also upgraded IVI guidance to Class Ia for stent implantation in the left main artery or complex lesions. However, the evidence is not uniformly positive across all clinical and lesion subsets. Recent randomized studies and meta-analyses have reported neutral findings in specific settings, including unprotected left main disease and complex high-risk PCI, highlighting ongoing uncertainty about which patient and lesion subsets derive the greatest incremental benefit from routine IVI guidance (11–13). Against this evolving evidence base and expanding guideline recommendations, longitudinal data on U.S. adoption trends and disparities remain sparse.

Using a nationally representative PCI cohort (2012–2022), we assessed temporal trends in IVI-guided PCI utilization, demographic and hospital disparities, and associated in-hospital outcomes and resource utilization. We hypothesized that adoption would increase but exhibit persistent disparities across hospital types and regions.

## Material and methods

### Data source

We conducted a retrospective cohort study using the National Inpatient Sample (NIS) (2012-2022), the largest nationally representative all-payer inpatient database in the United States, sponsored by the Agency for Healthcare Research and Quality (AHRQ) and maintained under the Healthcare Cost and Utilization Project (HCUP) (14). The NIS utilizes a stratified 20% sample of U.S. community hospitals, capturing over 100 clinical and non-clinical variables from approximately 7 million unweighted admissions annually; national estimates were generated using HCUP-supplied discharge weights.

### Study population

We identified hospitalizations with primary procedure codes for PCI. IVI-guided PCI was defined by concurrent codes for IVUS (ICD-9-CM 00.24; ICD-10-PCS B241ZZ3, B240ZZ3) or OCT (ICD-9-CM 00.25; ICD-10-PCS B221Z2Z, B223Z2Z) (15–17). Diagnosis, procedure, comorbidity, and in-hospital outcome codes are listed in Supplementary Table S1. Patients younger than 18 years or with missing mortality data were excluded. Total charges were merged with cost-to-charge ratio files to calculate hospital costs. The use of the NIS database to describe the prevalence and outcomes of cardiovascular disease has been previously validated (18, 19). Institutional review board approval was not sought due to the publicly available nature of this deidentified database. These data are accessible via HCUP-NIS. The authors vouch for the data's accuracy and completeness.

**Table 1:** Baseline characteristics of cohorts stratified by intravascular imaging use in PCI treated hospitalizations.

Variables	PCI without IVI guidance *n* = 4,715,995	IVI-guided PCI *n* = 504,595	*p*-Value	*SMD*
Age	65 (57–74)	66 (57–75)	<0.001	0.019
Female	1,544,830 (32.76)	161,480 (32.00)	<0.001	0.016
AMI	3,300,535 (69.99)	329,205 (65.24)	<0.001	0.102
Cardiogenic shock	263,980 (5.60)	28,540 (5.66)	0.09	0.003
Cardiac arrest	167,415 (3.55)	17,480 (3.46)	0.003	0.005
Race			<0.001	
White	3,412,405 (75.66)	367,092 (72.75)		0.067
Black	431,920 (9.58)	43,965 (9.14)		0.015
Hispanic	363,710 (8.06)	54,783 (10.86)		0.060
Asian/Pacific islander	119,525 (2.65)	15,020 (3.12)		0.028
Native American	25,490 (0.57)	3,475 (0.72)		0.019
Other races	156,910 (3.48)	20,260 (4.21)		0.038
Hypertension	3,686,580 (78.17)	401,475 (79.56)	<0.001	0.034
Diabetes mellitus	1,892,680 (40.13)	202,415 (40.11)	0.80	0.000
Atrial fibrillation	703,875 (14.93)	81,240 (16.10)	<0.001	0.032
History of smoke	1,673,605 (35.49)	173,330 (34.35)	<0.001	0.024
Obesity	949,860 (20.14)	108,635 (21.53)	<0.001	0.034
Prior MI	788,725 (16.72)	95,205 (18.87)	<0.001	0.056
Prior PCI	947,110 (20.08)	108,200 (21.44)	<0.001	0.034
Prior CABG	416,505 (8.83)	36,570 (7.25)	<0.001	0.058
Prior stroke	317,215 (6.73)	35,610 (7.06)	<0.001	0.013
Peripheral arterial disease	518,495 (10.99)	60,180 (11.93)	<0.001	0.030
Chronic lung disease	891,665 (18.91)	98,060 (19.43)	<0.001	0.013
Hospital size (number of beds)			<0.001	
Small	669,740 (14.20)	65,825 (13.05)		0.034
Medium	1,341,794 (28.45)	128,575 (25.48)		0.067
Large	2,704,461 (57.35)	310,195 (61.47)		0.084
Hospital location/teaching status			<0.001	
Rural hospital	279,610 (5.93)	25,925 (5.14)		0.035
Urban non-teaching	1,178,090 (24.98)	103,900 (20.59)		0.105
Urban teaching	3,258,294 (69.09)	374,770 (74.27)		0.115
Hospital location			<0.001	
Northeast	808,735 (17.15)	91,800 (18.19)		0.027
Midwest	1,123,770 (23.83)	118,170 (23.42)		0.010
South	1,984,940 (42.09)	169,600 (33.61)		0.176
West	798 550 (16.93)	125,025 (24.78)		0.194
Payer			<0.001	
Medicare	2,504,065 (53.19)	270,520 (53.68)		0.010
Medicaid	420,835 (8.94)	47,550 (9.44)		0.017
Private	1,367,100 (29.04)	142,275 (29.22)		0.004
Self-pay	246,595 (5.24)	21,300 (4.23)		0.048
No charge	24,175 (0.51)	1,475 (0.29)		0.035
Other	145,445 (3.09)	15,850 (3.15)		0.003

Values *n* (%) or median (interquartile range).

AMI, acute myocardial infarction; CABG, coronary artery bypass grafting; PCI, percutaneous coronary intervention; MI, myocardial infarction.

### Statistical analyses

Continuous variables were summarized as medians with interquartile ranges (IQRs), and categorical variables as frequencies and percentages. NIS discharge weights were applied to generate national estimates. Baseline differences between IVI and non-IVI PCI groups were assessed using standardized mean differences (SMDs), with an absolute SMD >0.10 considered clinically meaningful.

Temporal trends were evaluated using multivariable logistic regression with 2012 as the reference year. The association between IVI and in-hospital mortality was examined using survey-weighted multivariable logistic regression, adjusting for age, sex, race, STEMI, NSTEMI, cardiogenic shock, cardiac arrest, cardiovascular comorbidities, payer, hospital size, hospital location/teaching status, hospital region, and calendar year. Adjusted odds ratios (aORs) with 95% confidence intervals (CIs) were reported. Prespecified subgroup analyses were performed by sex, race, hospital size, hospital region, age group, and hospital location/teaching status, using interaction terms to assess heterogeneity across subgroups.

Propensity score matching was conducted to further address confounding by indication. Patients were matched 1:1 using nearest-neighbor matching without replacement, with a caliper of 0.2 standard deviations of the propensity score and exact matching on calendar year. Covariate balance was assessed using absolute SMDs, with values <0.10 indicating adequate balance. In-hospital mortality was subsequently compared between the matched groups.

All tests were two-sided, with *P* < 0.05 considered statistically significant. Analyses were performed using SAS version 9.4 (SAS Institute, Cary, NC, USA) and R 4.6.1 (R Foundation for Statistical Computing).

### Study outcomes

Primary outcomes of interest include the temporal adoption trends in the percentages of IVUS-guided intravascular guided PCIs from 2012 to 2022 and demographic and hospital disparities. Secondary outcomes include trends in in-hospital mortality, length of stay (LOS), and total hospital cost.

## Results

### National cohort characteristics

Among 5,220,590 PCI procedures during 2012–2022, 504,595 (9.67%) utilized IVI guidance (485,005 with IVUS and 22,145 with OCT). Key characteristics are shown in Table 1. The median age was 65 years [IQR 57–74] without IVI vs. 66 [IQR 57–75] with IVI, and female representation was comparable (32.76% vs. 32.00%). Most IVI-guided PCIs occurred in large hospitals (61.67%) and urban teaching hospitals (74.27%).

### Temporal trends in IVI adoption

IVI-guided PCI increased threefold from 7.05% in 2012 to 21.25% in 2022) (*p*_for trend_ < 0.001) (Figure 1A). The combination of physiology and IVI guidance for PCI procedures remained low but rose from 0.26% in 2012 to 1.47% in 2022 (*p*_for trend_ < 0.001). Compared with 2012, the adjusted odds ratio (aOR) for IVI use in 2022 was 3.06 (95% CI: 2.89-3.24); for combined IVI and physiology guidance, aOR = 5.59 (95% CI: 4.62–6.77) (Figure 1B).

**Figure 1:**
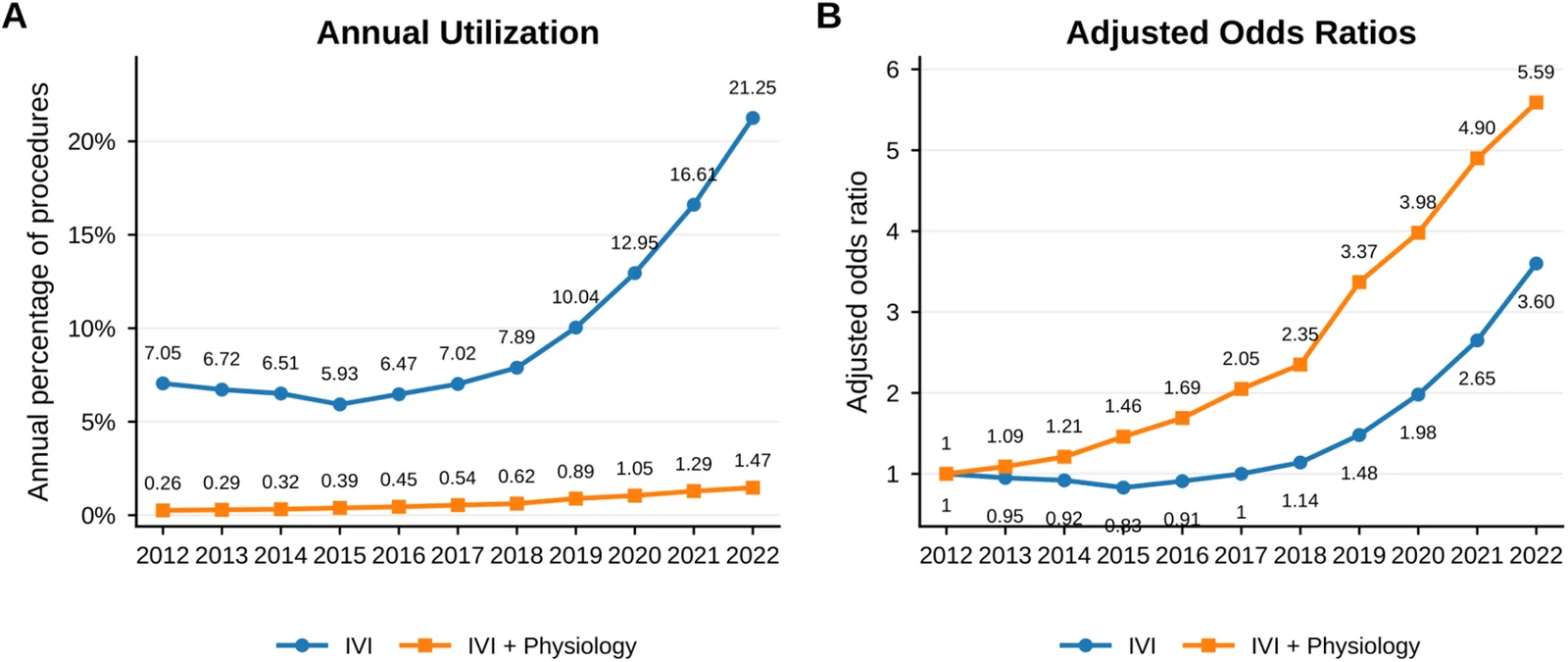
**(A)** Annual percentages of IVI-guided and IVI+physiology-guided PCI in total PCI. **(B)** Adjusted odds ratios for IVI-guided and IVI+physiology-guided PCI.

### Disparities in IVI adoption

Minimal sex differences existed, with near-identical adoption in 2012 (female 6.99% vs. male 7.19%) and 2022 (female 21.51% vs. male 20.70%) (Figure 2A). Similarly, the percentage of IVI-guided PCI procedures was comparable between white and non-white cohorts throughout the study period (Figure 2B). However, pronounced hospital-level disparities were observed, with a striking gradient emerging by location and hospital teaching status. In 2022, IVI-guided PCI was 15.47% for rural hospitals, 18.73% for urban non-teaching hospitals, and 22.22% for urban teaching hospitals (*p* < 0.001 across groups) (Figure 2C). There were persistent regional inequities, with rates of 6.03%, 6.61%, 6.75%, and 10.46% in 2012, and 16.28%, 22.24%, 25.05%, and 27.62% in 2022 for the West, South, Midwest, and Northeast, respectively (Figure 2D).

**Figure 2:**
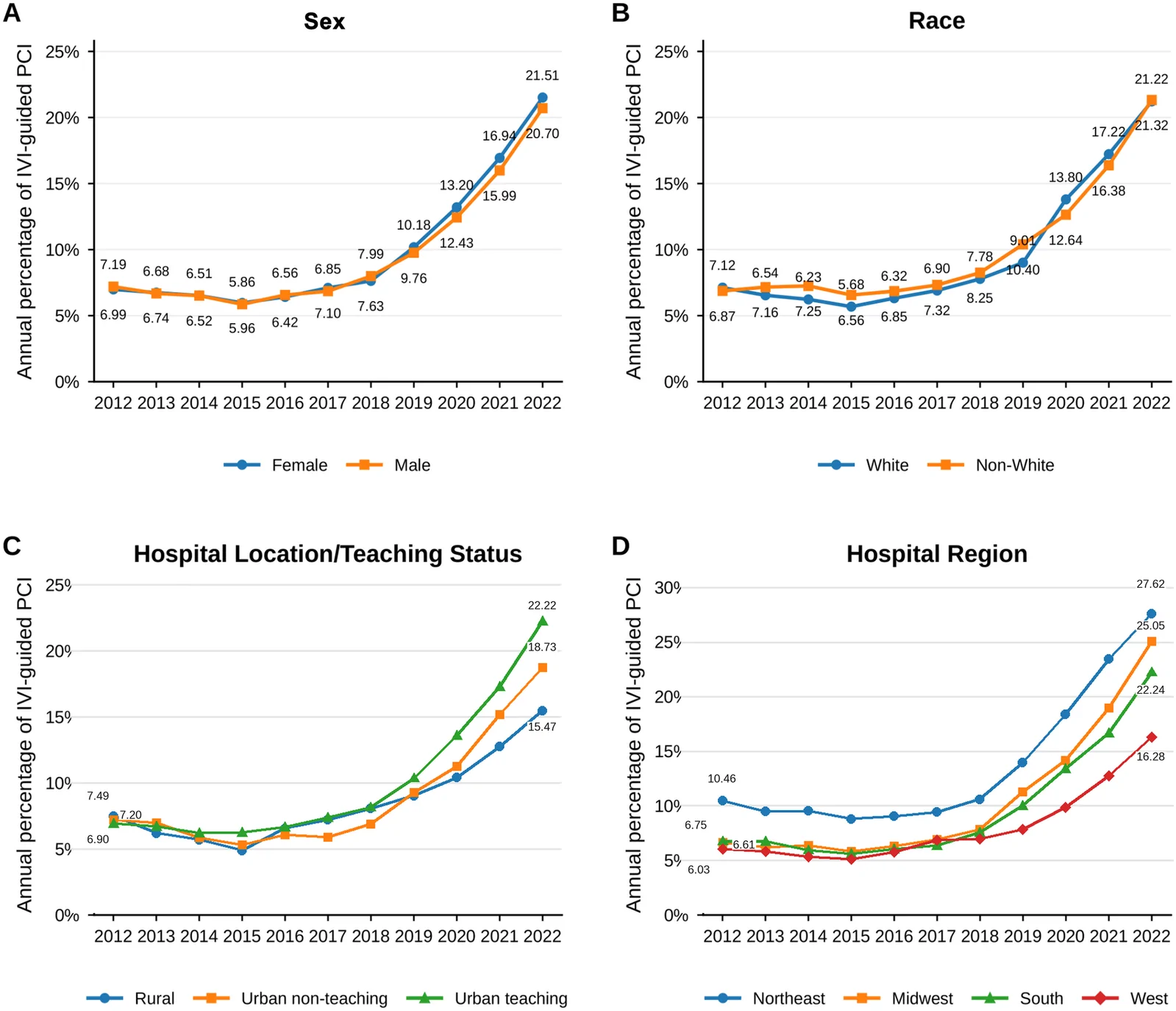
Annual percentages of IVI-guided PCI in total PCI stratified by (**A**) sex, (**B**) race, (**C**) hospitallocation/teaching status, and (**D**) hospita lregion.

### Clinical outcomes and resource utilization

IVI-guided PCI was associated with lower mortality compared to angiography-guided PCI (2.32% vs. 2.88%, aOR 0.78, 95% CI 0.74–0.83; *p* < 0.001). There was also a higher routine home discharge rate (84.12% vs. 83.71%; *p* < 0.001). However, IVI guidance incurred modestly higher resource use, including a longer LOS: median 3 days [IQR 2–5] vs. 2 days [IQR 2–4]; *p* < 0.001, and higher hospital costs ($25,144 [IQR $18,749–$36,439] vs. $20,165 [IQR $15,293–$28,381]; *p* < 0.001) (Table 2).

**Table 2:** Clinical outcomes of cohorts stratified by intravascular imaging.

Outcome	PCI without IVI guidance *n* = 4,715,995	IVI-guided PCI *n* = 504,595	*p*-Value
In-hospital mortality	135,980 (2.88)	11,700 (2.32)	<0.001
Discharge status			<0.001
Home	3,725,700 (83.71)	400,405 (84.12)	
Transfer to other hospital	52,130 (1.17)	4,300 (0.90)	
Skilled nursing facility	246,020 (5.53)	23,960 (5.03)	
Home healthcare	340,150 (7.64)	39,400 (8.28)	
Length of stay, days	2 (2–4)	3 (2–5)	<0.001
Mean cost, $	20,165 (15,293–28,381)	25,144 (18,749–36,439)	<0.001

Values *n* (%) or median (interquartile range).

### Subgroup analysis

The mortality reduction with IVI was consistent across all subgroups (Figure 3): male (aOR 0.78, 95% CI 0.73–0.84) vs. female (aOR 0.78, 95% CI: 0.72–0.86); white (aOR 0.77, 95% CI: 0.73–0.83) vs. non-white (aOR 0.79, 95% CI: 0.71–0.89); age <50 years (aOR 0.61, 95% CI: 0.48–0.78), 50y-75y (aOR 0.78, 95% CI: 0.72–0.84） ≥ 75 years (aOR 0.81, 95% CI: 0.74–0.88), rural hospital (aOR 0.78, 95% CI: 0.60–1.02), urban non-teaching (aOR 0.73, 95% CI: 0.64–0.83), urban teaching (aOR 0.80, 95% CI: 0.75–0.85) (*p*_for interaction_ > 0.05 for all subgroups). The study selection process is summarized in Figure 4.

**Figure 3:**
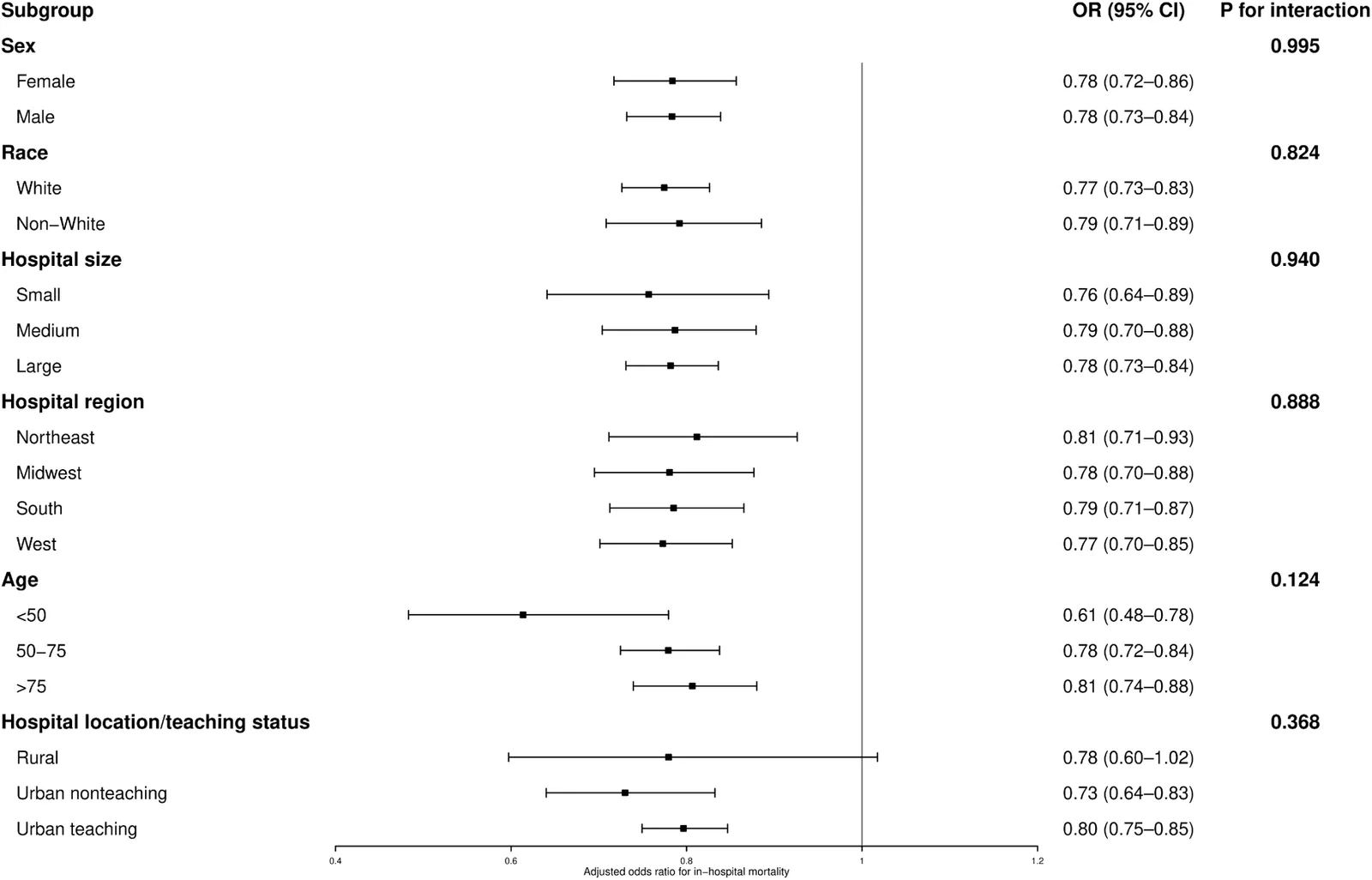
Forest plot of in-hospital mortality for the subgroup analysis.

**Figure 4:**
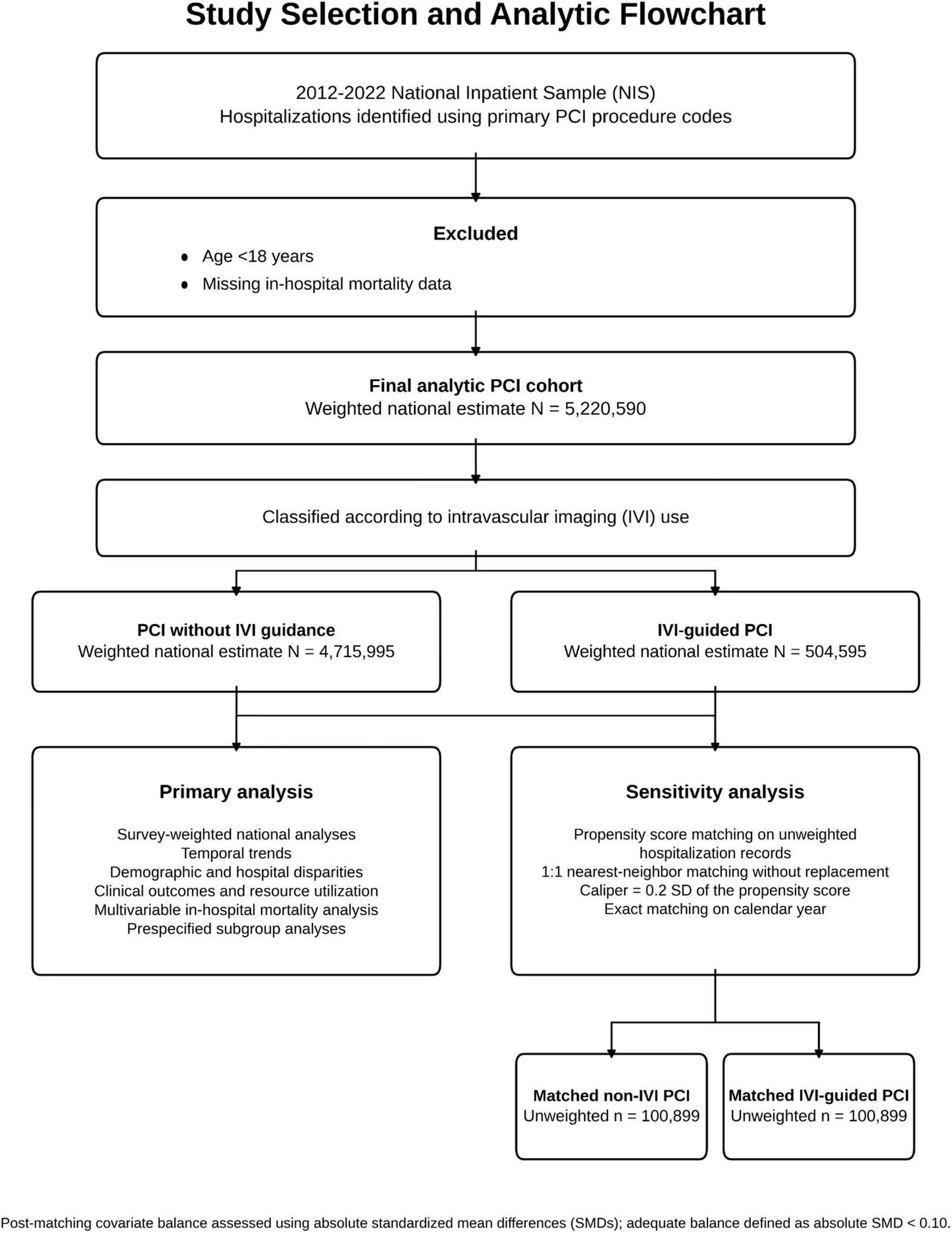
Study selection flowchart.

## Discussion

Our study demonstrates accelerated IVI adoption in contemporary U.S. practice (9.67% overall; 21.25% in 2022), contrasting with earlier reports of 4.8% (2004–2014) (20). Interestingly, the adoption of IVI-guided procedures showed a non-linear trajectory, stagnating at around 7% (2012–2017) before nearly tripling (2018–2022). This surge aligns with pivotal evidence and guideline updates, including the landmark trials. The IVUS-XPL trial demonstrated a 52% reduction in 1-year MACE with IVUS-guided PCI for patients requiring long coronary stent implantation (1), and the ULTIMATE trial showed a 47% lower target vessel failure in all-comers (3). With recent Class Ia recommendations for IVI-guided PCI (ESC 2024 Chronic Syndromes and ACC/AHA 2025 ACS (9, 10)—particularly for left main, bifurcation, and complex lesions—adoption is expected to accelerate beyond our observed 21.3% in 2022.

While female and non-white patients historically experience significant inequities in cardiovascular care—including lower rates of guideline-directed medical therapy (21–23) and reduced PCI access (24, 25)—our study reveals a notable exception in IVI-guided PCI adoption.

We observed nearly identical adoptions in 2012 (female: 6.99% vs. male: 7.15%) and parallel acceleration over the study period. (*p* = 0.78), with statistically indistinguishable adoption rates for white and non-white individuals at all time points and equivalent relative increases across racial groups. Unlike other cardiovascular interventions, IVI adoption shows no sex or racial bias—a model for equitable technology diffusion.

However, significant disparities emerged across hospital types and regions (20). Remarkably, the 2018–2022 surge was primarily driven by urban teaching hospitals, widening rural–urban gaps despite universal mortality benefits*.* The 6.75% urban–rural gap represents over 32,000 fewer IVI-guided PCI procedures in rural hospitals in 2022, a critical inequity given IVI's mortality benefit (8). The Northeast region showed the highest adoption rate throughout the study period, correlating with a higher density of urban teaching hospitals, while western states faced unique geographic barriers.

IVI was more commonly used at large urban teaching hospitals, likely due to higher PCI volumes, greater operator experience, improved access to advanced imaging technologies, comprehensive multidisciplinary expertise, and standardized peri-procedural care pathways. Importantly, these institutional characteristics may affect both the likelihood of IVI use and clinical outcomes. Although our multivariable models adjusted for a wide range of patient and hospital-level characteristics, the NIS lacks detailed information on operator volume and experience, lesion complexity, procedural success, stent optimization, and institution-specific PCI protocols. Therefore, residual confounding from these unmeasured factors may partly explain the observed association between IVI use and lower in-hospital mortality. Additionally, the lower adoption of IVI in rural and non-teaching hospitals highlights a significant implementation gap. Limited institutional resources, training opportunities, and geographic access may contribute to these disparities. Addressing this gap may require targeted strategies to improve access to IVI technology and expertise, such as regional training networks, remote procedural support, and appropriate reimbursement mechanisms.

IVI was associated with a modestly longer length of stay and higher hospital costs. These differences likely reflect several contributing factors. IVI use requires additional imaging catheters, equipment, procedural time, and image acquisition and interpretation, which can increase resource utilization. Moreover, IVI may be preferentially used in more anatomically or procedurally complex cases, which can also lead to longer hospital stays and greater resource use.

In this study, IVI was associated with lower adjusted odds of in-hospital mortality, and this association was generally consistent across the prespecified subgroups. These findings align with prior studies suggesting favorable outcomes with imaging-guided PCI. However, recent randomized trials have reported neutral clinical outcomes in selected populations, including patients undergoing unprotected left main and complex high-risk PCI (11–13). Several mechanisms may explain the lower in-hospital mortality observed in our cohort. IVI can facilitate more accurate vessel sizing, optimize stent expansion and apposition, and identify procedural complications such as edge dissection or geographic miss that may not be apparent on angiography alone.

These findings carry important implications. First, although overall IVI use has increased in the United States, substantial differences across hospitals remain, indicating that greater national adoption does not guarantee equal access to this technology. Second, the relatively small differences in IVI use across sex and racial groups suggest that equitable adoption of new procedural technologies may be achievable. In contrast, hospital resources and technical capabilities may play a more significant role in determining access to IVI. Finally, while IVI use was associated with lower in-hospital mortality, it was also linked to greater resource utilization, and randomized trials have shown somewhat inconsistent results across different clinical settings. Therefore, future use of IVI may be best guided by patient and lesion characteristics rather than simply expanding its use across all PCI procedures.

Future research should more precisely define which patient and lesion subgroups derive the greatest incremental benefit from IVI, determine the relative efficacy and cost-effectiveness of IVUS and OCT across various anatomical and clinical scenarios, and validate whether routine imaging can improve outcomes in low-complexity PCI procedures. In particular, pragmatic randomized controlled trials and prospective registry studies are needed, incorporating variables such as lesion morphology, procedural optimization metrics, operator and hospital PCI volume, and longitudinal outcomes. Additionally, implementation studies should assess whether structured training programs, changes in insurance reimbursement policies, regional referral networks, and standardized imaging protocols can reduce the inter-hospital disparities observed in this study.

### Limitations

NIS lacks granular data on lesion complexity such as SYNTAX score, calcification, bifurcation, and procedural details like stent expansion, malapposition, medical therapies, and laboratory values. Moreover, there were no long-term follow-up data in the NIS dataset. Coding errors and underreporting of secondary diagnoses are also potential sources of bias. Despite multivariable adjustment, residual confounding, such as operator experience and hospital PCI volume, may influence outcomes. However, the NIS database has been extensively validated in previous publications. This study included the largest sample of hospitalized patients who underwent PCI procedures, bridging an important knowledge gap by reporting the demographic and hospital disparities in real-world practice. This study provides the first national quantification of hospital-level IVI disparities—a prerequisite for equity-focused intervention.

## Conclusion

While IVI-guided PCI adoption increased significantly, utilization remains low with pronounced hospital-level disparities. Demographic equity proves achievable, yet institutional gaps demand urgent solutions. Future guidelines must pair Class Ia recommendations with disparity-mitigation strategies to ensure life-saving technologies reach all patients—not only those in academic centers.

## Clinical perspective

What is new?

IVI-guided PCI adoption showed a non-linear trajectory during the study period, 2012–2017, with stagnation: Rates plateaued at around 7% despite early evidence, followed by a surge from 2018–2022. Despite the overall increase in IVI-guided PCI adoption rates, significant hospital disparities persist, with few demographic disparities observed.

What are the clinical implications?

IVI confers a universal mortality benefit, yet access inequities limit population-level impact. Prioritizing hospital-level interventions (reimbursement reform, simulation training, telehealth) is essential for standardizing PCI quality nationwide.

## Data Availability

The raw data supporting the conclusions of this article will be made available by the authors, without undue reservation.
