# Supplementary material for: Adoption of intravascular imaging-guided percutaneous coronary intervention in the United States (2012–2022): temporal trends, disparities, and mortality association
Source: Front Cardiovasc Med. 2026 Oct 7;13:1930050. doi: 10.3389/fcvm.2026.1930050 (PMC13684127; doi:10.3389/fcvm.2026.1930050)
Supplement: Supplementary file 1 [file Table1.docx]

| Variables | Code type | ICD 9 | ICD10 |
| --- | --- | --- | --- |
| IVUS | PR | 0024 | B241ZZ3 B240ZZ3 |
| OCT | PR | 3824 | B221Z2Z B223Z2Z |
| FFR | PR | 0059 | 4A033BC |
| PCI | PR | 0066 3606 3607 | 0210344 02103D4 0211344 02113D4 02123D4 0270346 027034Z 0270356 027035Z 0270366 027036Z 0270376 027037Z 02703D6 02703DZ 02703E6 02703EZ 02703F6 02703FZ 02703G6 02703GZ 02703T6 02703TZ 02703Z6 02703ZZ 0271346 027134Z 0271356 027135Z 0271366 027136Z 0271376 027137Z 02713D6 02713DZ 02713E6 02713EZ 02713F6 02713FZ 02713G6 02713GZ 02713T6 02713TZ 02713Z6 02713ZZ 0272346 027234Z 0272356 027235Z 0272366 027236Z 0272376 027237Z 02723D6 02723DZ 02723EZ 02723F6 02723FZ 02723G6 02723GZ 02723TZ 02723Z6 02723ZZ |
| Angio | PR | 8853 8854 8855 8856 8857 3722 3723 | B2050ZZ B2051ZZ B205YZZ B2150ZZ B2151ZZ B215YZZ |
| STEMI | DX | 41000 41001 41002 41010 41011 41012 41020 41021 41022 41030 41031 41032  41040 41041 41042 41050  41051 41052 41060 41061  41062 41080 41081 41082  41090 41091 41092 | I2101 I2102 I2109 I2111 I2119 I2121 I2129 I213 |
| Non-STEMI | DX | 41070 41071 41072 | I214 |
| Atrial fibrillation | DX | 42731 | I480 I481 I4811 I4819 I482 I4820 I4821 I4891 |
| Acute kidney injury | DX | 5845 5846 5847 5848 5849 | N170 N171 N172 N178 N179 N19 N990 R34 R944 |
| Cardiogenic shock | DX | 78551 | R570 |
| Cardiac arrest | DX | 4275 | I469 I462 I468 I97120 I97121 I97130 I97710 |
| Smoke history | DX | V1582 3051 | Z87891 F17200 F17201 F17203 F17208 F17209 F17210 F17211 F17213 F17218 F17219 F17220 F17221 F17223 F17228 F17229 F17290 F17291 F17293 F17298 F17299 Z720 |
| Prior myocardial infarction | DX | 412 | I252 |
| Prior CABG | DX | V4581 | Z951 |
| Prior PCI | DX | V4582 | Z9861 Z955 |
| Family history of CAD | DX | V1254 | Z8248 Z8241 |
